# Parental pre-pregnancy body mass index and risk of low birth weight in offspring: A prospective cohort study in central China

**DOI:** 10.3389/fpubh.2022.1036689

**Published:** 2022-11-30

**Authors:** Jianhui Wei, Tingting Wang, Jing Shu, Yiping Liu, Xinli Song, Mengting Sun, Taowei Zhong, Qian Chen, Manjun Luo, Senmao Zhang, Peng Huang, Ping Zhu, Donghua Xie, Jiabi Qin

**Affiliations:** ^1^Department of Epidemiology and Health Statistics, Xiangya School of Public Health, Central South University, Changsha, China; ^2^Hunan Institute of Reproductive Medicine, Hunan Provincial Maternal and Child Health Care Hospital, Changsha, China; ^3^Department of Cardiothoracic Surgery, Hunan Children's Hospital, Changsha, China; ^4^Guangdong Cardiovascular Institute, Guangdong Provincial People's Hospital, Guangdong Academy of Medical Sciences, Guangzhou, China; ^5^Hunan Provincial Key Laboratory of Clinical Epidemiology, Changsha, China

**Keywords:** pre-pregnancy, body mass index, low birth weight, risk factor (RF), cohort study [or longitudinal study]

## Abstract

**Background:**

Low birth weight (LBW) is one of the most common adverse pregnancy outcomes. Previous studies have consistently shown that maternal body mass index (BMI) status before and during pregnancy is associated with LBW. However, previous studies lacked an association between paternal BMI and the conjunction effect of a couple's BMI and LBW in the offspring. Therefore, we established a cohort of pre-pregnancy couples to prospectively assess the relationship between maternal and paternal pre-pregnancy BMI and offspring LBW, very low birth weight (VLBW), and extremely low birth weight (ELBW).

**Methods:**

A prospective cohort study was established in Central China. A total of 34,104 pregnant women with singleton pregnancies at 8–14 gestational weeks and their husbands were finally enrolled and followed to 3 months postpartum. The multivariate logistic regression and restrictive cubic spline model were used to explore the relationship between parental pre-pregnancy BMI and the risk of LBW, VLBW, and ELBW in offspring.

**Results:**

Of the 34,104 participants, maternal pre-pregnancy overweight and obesity were associated with a higher risk of LBW (overweight: OR = 1.720, 95% CI = 1.533 ~ 1.930; obesity: OR = 1.710, 95% CI = 1.360 ~ 2.151), VLBW (overweight: OR = 2.283, 95% CI = 1.839 ~ 2.834; obesity: OR = 4.023, 95% CI = 2.855 ~ 5.670), and ELBW (overweight: OR = 3.292, 95% CI = 2.151 ~ 5.036; obesity: OR = 3.467, 95% CI = 1.481 ~ 8.115), while underweight was associated with a higher risk of LBW (OR = 1.438, 95% CI = 1.294 ~ 1.599) and a lower risk of ELBW (OR = 0.473, 95% CI = 0.236 ~ 0.946). Paternal pre-pregnancy overweight and obesity were associated with a higher risk of LBW (overweight: OR = 1.637, 95% CI = 1.501 ~ 1.784; obesity: OR = 1.454, 95% CI = 1.289 ~ 1.641) and VLBW (overweight: OR = 1.310, 95% CI = 1.097 ~ 1.564; obesity: OR = 1.320, 95% CI = 1.037 ~ 1.681), while underweight was associated with a lower risk of LBW (OR = 0.660, 95% CI = 0.519 ~ 0.839). Parents who were both excessive-weights in pre-pregnancy BMI, as well as overweight mothers and normal-weight fathers before pre-pregnancy, were more likely to have offspring with LBW, VLBW, and ELBW. Dose-response relationship existed between parental pre-pregnancy and LBW, VLBW, and ELBW, except for paternal BMI and ELBW.

**Conclusions:**

Parental pre-pregnancy BMI was associated with the risk of LBW in offspring. Management of weight before pregnancy for couples might help reduce their adverse pregnancy outcomes in future intervention studies.

## Introduction

Low birth weight (LBW) is defined as a newborn birth weight of < 2,500 g and is one of the common adverse pregnancy outcomes (APO), it includes very low birth weight (birth weight < 1,500 g) and extremely low birth weight (birth weight < 1,000 g) (1). LBW is estimated to be about 20 million births per year globally, accounting for 15–20% of all births worldwide (2). The worldwide LBW prevalence is ~14.6%, 91% of which are from low-and-middle-income countries (3), and in China, the overall prevalence of LBW is 5.15%, with 4.57% in boys and 5.68% in girls (4). The impact of LBW on health-related quality of life is long-term: compared to normal individuals, infants with LBW have an elevated risk of mortality in the first year of life (5) and perform worse than their peers in the pre-school years in terms of physical, emotional and/or social functioning (6); during compulsory education, children born with LBW present with the disease of the nervous system and mental and behavioral disorders, some of the effects which persist into early adulthood (7); and in adulthood, they are at higher risk of respiratory disease (8), diabetes (9), hypertension (10), coronary heart disease (11)in adulthood.

Maternal weight or BMI before pregnancy can be considered an indicator of maternal nutritional status, and maternal nutritional status can reduce placental-fetal blood flows and stunt fetal growth (12), many studies have explored the relationship between maternal abnormal pre-pregnancy BMI and LBW as well as other adverse pregnancy outcomes (13, 14). Along with the “Developmental Origins of Health and Disease” (DOHaD) theory, the “Paternal Origins of Health and Disease” (POHaD) theory has also been proposed, and the epigenome of sperm cells is indeed influenced by paternal exposure, which subsequently affects male fertility and offspring health (15, 16), therefore we need to focus more on the contribution of paternal factors to offspring health. Much of the current research has focused on the effect of maternal BMI on pregnancy outcomes, while studies on the relationship between preconception paternal or couple BMI and LBW and other APOs are scarce and contradictory (17, 18). Some studies have suggested that male obesity is associated with reduced reproductive potential (19) and abnormal weight reduces semen quality (20). Similarly, obesity induces ovarian inflammation and reduces oocyte quality, negatively affecting female fertility (21, 22). Since pregnancy failure is influenced by a combination of biological and social factors of both partners, prevention of LBW should begin with early intervention of risk factors in both spouses before pregnancy, and it is essential to study the effect of BMI on LBW in both spouses before pregnancy and to develop a reasonable weight control plan from the time of pregnancy preparation.

Given the high risk of LBW in newborns and the lack of clarity regarding the association of both husband and wife, especially paternal factors with LBW, it is essential to clarify their association. To this end, in this study, we aimed to assess the association between preconception BMI of both spouses and LBW in the offspring in a prospective cohort study to support counseling guidelines aimed at optimizing preconception health and pregnancy outcomes.

## Methods

### Participants

Data were collected from a prospective cohort study conducted at the Hunan Provincial Maternal and Children Health Care Hospital, the provincial health center for women and children in Hunan Province, Central China. From March 2013 to December 2019, pregnant women (≥18 years) who had their first antenatal visit at the study hospital during 8–14 weeks of gestation and intended to continue antenatal care throughout their pregnancy and their husbands were enrolled in this study. After written informed consent, 40,650 pregnant women and their husbands were eventually included in the cohort study. Data from the last menstrual periods of the pregnant women were used to estimate the gestational week, and the ultrasound was used to calculate if the menstruation was irregular (23). Trained physicians conducted face-to-face interviews to obtain socio-demographic characteristics, gynecological data, obstetric data, health-related information of pregnant women in the cohort, and sociodemographic characteristics and health-related information of husbands. All included participants will be followed up until 3 months postpartum to obtain detailed information about their birth status. Exclusion criteria were: (i) artificial fertilization; (ii) multiple pregnancy; (iii) termination of pregnancy by abortion or induction of labor due to unintended pregnancy or ectopic pregnancy.

The study received approval from the Ethics Committee of Xiangya School of Public Health Central South University (No: XYGW-2018-36) and was conducted based on the Helsinki declaration. The study protocol has been registered with the Chinese Clinical Trial Registry (No: ChiCTR1800016635). We obtained written informed consent from all participants.

### Data collection

At the time of study population inclusion, we obtained sociodemographic data, disease history, pregnancy history records, and personal behavioral history of the pregnant women through study-specific questionnaires, as well as sociodemographic characteristics and personal behavioral characteristics of the husbands. In addition, we the height and weight of pregnant women at their first antenatal visit and their weight recorded at their last antenatal visit (data from the hospital's electronic medical record system). At 3 months postpartum, we obtained maternal outcomes, including gestational weeks, mode of delivery and maternal complications, and neonatal outcomes, including basic neonatal information and pregnancy outcomes, through the electronic medical record system and follow-up.

### Assessments of exposure

Body mass index was computed as body weight in kilograms divided by body height in meters squared. Based on the Chinese adult population BMI classification standards (24), BMI was classified into four categories: underweight (< 18.5 kg/m^2^), normal weight (18.5 ~ 23.9 kg/m^2^), overweight (24.0–27.9 kg/m^2^), and obesity (≥28 kg/m^2^). Pre-pregnancy BMI of pregnant women and their husbands using self-reported pre-pregnancy height and weight, obtained from questionnaire records at baseline survey. In addition, we obtained the maternal height and weight at the first and last antenatal visit to measure and calculate the weight gain during pregnancy using a ruler and a calibrated electronic scale, with the mother wearing light clothing but no shoes at all the time of measurement.

### Assessments of outcomes

Low birth weight was defined as an infant whose birth weight is < 2,500 g, regardless of gestational age. To further explore the relationship between parental BMI and low birth weight, we further divided low birth weight into very low birth weight, which is defined as < 1,500 g, and extremely low birth weight, which is defined as < 1,000 g. All LBW cases were diagnosed through obstetric specialists.

### Assessments of covariates

Covariates were selected based on a priori knowledge and clinical plausibility. Available potential confounders were maternal age, divided into four groups: < 25, 25–29, 30–34, and ≥35 years, ethnicity was classified into Han and other minorities, educational level was classified as junior high school or below, high school or technical school, junior school, and bachelor and above, parity (i.e., yes or no), family income per month was classified as ≤ 2,500, 2,500–5,000, and >5,000 RMB, active smoking before pregnancy (i.e., yes or no), passive smoking before pregnancy (i.e., yes or no), alcohol consumption before pregnancy (i.e., yes or no), folic acid consumption before or during pregnancy (i.e., yes or no), history of adverse pregnancy outcomes defined as a history of at least one of three: stillbirth, LBW and neonatal deaths, history of pregnancy complications defined as a history of at least one of two: gestational diabetes or hypertension of pregnancy, gestational weight gain recommendation rage classified into three groups: inadequate GWG group, adequate GWG group, and excessive GWG group, under following the 2009 Institute of Medicine (IOM) guideline (25), pregnancy complications in this pregnancy defined as at least one of two: gestational diabetes or hypertension of pregnancy in this pregnancy. The division of paternal characteristics is the same as that of maternal characteristics, paternal age was divided into four groups: < 25, 25–29, 30–34, and ≥35 years, ethnicity was classified into Han and other minorities, educational level was classified as junior high school or below, high school or technical school, junior school, and bachelor and above, smoking status before wife pregnancy (i.e., yes or no) and alcohol consumption before wife pregnancy (i.e., yes or no). All of which may affect both exposures and outcomes.

### Statistical analyses

Data were collated and analyzed using EpiData version 3.1 (EpiData Association, Odense, Denmark) and SAS OnDemand for Academics (SAS Institute Inc., Cary North Carolina, USA). The figures were plotted in R software (version 4.1.2). Descriptive statistics were used to assess the characteristics of the study population, and the Chi-square test was used to compare categorical variables. Association between parental BMI and low birth weight was assessed by univariable logistic regression analyses and multivariable logistic regression after adjusting for covariates. The conjunction effect between maternal BMI and paternal BMI with low birth weight was evaluated by crossover analysis, and based on the crossover analysis, we further performed a stratified analysis and divided the population into subgroups by maternal BMI category, and performed logistic regression to explore the relationship between paternal BMI and LBW in different maternal BMI-level populations. In addition, we used restricted cubic spline models fitted for logistic regression with 3-knots at the 10th, 50th, and 90th percentiles of BMI to plot the relationship between parental BMI as a continuous variable (rather than BMI categories) and LBW. The significance level of all tests was *P* < 0.05 (two-tailed).

## Results

### Characteristics of participants

From March 13, 2013, to December 31, 2019, 40,650 eligible couples were included in the cohort. After considering the inclusion and exclusion criteria, 34,104 eligible couples were eventually included for analysis (Supplementary Figure 1). For the mothers included in the study, 14.4% (*n* = 4,920) were underweight, 70.2% (*n* = 23,925) were normal weight, 12.7% (*n* = 4,334) were overweight, and 2.7% (*n* = 925) were obesity. There were statistically significant differences across four maternal BMI groups for maternal age, ethnicity, educational level, parity, history of adverse pregnancy outcomes, gestational weight gain recommendation rage, pregnancy complications in this pregnancy, and husband smoking status before wife pregnancy (all *P* < 0.05; Table 1). For fathers, 4.2% (*n* = 1,442) were underweight, 53.1% (*n* = 18,118) were normal weight, 31.1% (*n* = 10,592) were overweight, and 11.6% (*n* = 3,952) were obesity. There were statistically significant differences across four paternal BMI groups for paternal age, ethnicity, educational level, smoking status before wife pregnancy, alcohol consumption before wife pregnancy, family income per month, and wife's age, active smoking before pregnancy, alcohol consumption before pregnancy, history of adverse pregnancy outcomes, history of pregnancy complications, gestational weight gain recommendation rage, pregnancy complications in this pregnancy (all *P* < 0.05; Table 2).

**Table 1:** The distribution of baseline characteristics of mothers enrolled in the cohort by categories of BMI (kg/m^2^).

**Characteristic**	** < 18.5 Underweight (%)**	**18.5 ~23.9** **Normal weight (%)**	**24.0 ~27.9 Overweight (%)**	**≥28.0 Obesity (%)**	***P*-value**
*n*	4,920 (14.4)	23,925 (70.2)	4,334 (12.7)	925 (2.7)	
**Age**
< 25 y	481 (27.2)	1,075 (60.8)	172 (9.7)	41 (2.3)	< 0.001
25 ~ 29 y	2,290 (19.3)	8,156 (68.7)	1,195 (10.1)	232 (2.0)	
30 ~ 34 y	1,607 (12.6)	9,066 (70.8)	1,760 (13.7)	370 (2.9)	
≥35 y	542 (7.1)	5,628 (73.5)	1,207 (15.8)	282 (3.7)	
**Ethnicity**
Han	4,871 (14.5)	23,601 (70.1)	4,285 (12.7)	899 (2.7)	< 0.001
Minority	49 (10.9)	324 (12.3)	49 (10.9)	26 (5.8)	
**Educational level**
Junior high school or Below	367 (14.4)	1,572 (61.7)	495 (19.4)	113 (4.4)	< 0.001
High school or Technical school	1,338 (13.8)	6,599 (68.1)	1,407 (14.5)	351 (3.6)	
Junior collage	2,399 (15.4)	11,091 (71.1)	1,800 (11.5)	304 (2.0)	
Bachelor and above	816 (13.0)	4,663 (74.4)	632 (10.1)	157 (2.5)	
**Parity**
Yes	1,936 (11.0)	12,593 (71.3)	2,589 (14.7)	540 (3.1)	< 0.001
No	2,984 (18.1)	11,332 (68.9)	1,745 (10.6)	385 (2.3)	
**Family income per month**
≤ 2,500 RMB	825 (14.0)	4,126 (70.0)	778 (13.2)	163 (2.8)	0.642
2,500–5,000 RMB	2,672 (14.7)	12,737 (70.0)	2,312 (12.7)	485 (2.7)	
>5,000 RMB	1,423 (14.2)	7,062 (70.6)	1,244 (12.4)	277 (2.8)	
**Active smoking before pregnancy**
Yes	43 (12.6)	255 (74.6)	36 (10.5)	8 (2.3)	0.355
No	4,877 (14.5)	23,670 (70.1)	4,298 (12.7)	917 (2.7)	
**Passive smoking before pregnancy**
Yes	690 (14.0)	3,517 (71.2)	620 (12.6)	111 (2.3)	0.091
No	4,230 (14.5)	20,408 (70.0)	3,714 (12.7)	814 (2.8)	
**Alcohol consumption before pregnancy**
Yes	91 (15.7)	385 (66.4)	87 (15.0)	17 (2.9)	0.222
No	4,829 (14.4)	23,540 (70.2)	4,247 (12.7)	908 (2.7)	
**Folic acid consumption before or during pregnancy**
Yes	4,708 (14.5)	22,807 (70.1)	4,146 (12.7)	895 (2.8)	0.134
No	212 (13.7)	1,118 (72.2)	188 (12.1)	30 (1.9)	
**History of adverse pregnancy outcomes**
Yes	856 (15.9)	3,659 (67.9)	710 (13.2)	163 (3.0)	< 0.001
No	4,064 (14.2)	20,266 (70.6)	364 (12.6)	762 (2.7)	
**History of pregnancy complications**
Yes	708 (15.5)	3,200 (70.1)	539 (11.8)	121 (2.6)	0.052
No	4,212 (14.3)	20,725 (70.2)	3,795 (12.8)	804 (2.7)	
**Gestational weight gain range**
Inadequate GWG group	1,638 (17.0)	7,393 (76.8)	505 (5.2)	93 (1.0)	< 0.001
Adequate GWG group	2,429 (16.1)	10,860 (71.8)	1,557 (10.3)	270 (1.8)	
Excessive GWG group	853 (9.1)	5,672 (60.6)	2,272 (24.3)	562 (6.0)	
**Pregnancy complications in this pregnancy**
Yes	616 (9.5)	4,421 (68.4)	1,129 (17.5)	299 (4.6)	< 0.001
No	4,304 (15.6)	19,504 (70.6)	3,205 (11.6)	626 (2.3)	
**Paternal characteristic**
**Age**
< 25 y	332 (14.2)	1,638 (70.3)	286 (12.3)	74 (3.2)	0.799
25 ~ 29 y	1,760 (14.3)	8,707 (70.5)	1,559 (12.6)	320 (2.6)	
30 ~ 34 y	1,644 (14.3)	8,047 (70.1)	1,468 (12.8)	317 (2.8)	
≥35 y	1,184 (14.9)	5,533 (69.6)	1,021 (12.8)	214 (2.7)	
**Ethnicity**
Han	4,760 (14.4)	23,142 (70.1)	4,196 (12.7)	902 (2.7)	0.614
Minority	160 (14.5)	783 (70.9)	138 (12.5)	23 (2.1)	
**Educational level**
Junior high school or Below	603 (14.6)	2,888 (69.8)	544 (13.1)	103 (2.5)	0.135
High school or Technical school	1,252 (14.4)	6,077 (69.7)	1,141 (13.1)	244 (2.8)	
Junior collage	2,640 (14.4)	12,927 (70.4)	2,317 (12.6)	478 (2.6)	
Bachelor and above	425 (14.7)	2,033 (70.3)	332 (11.5)	100 (3.5)	
**Smoking status before wife pregnancy**
Yes	2,112 (14.5)	10,096 (69.4)	1,953 (13.4)	397 (2.7)	0.006
No	2,808 (14.4)	13,829 (70.8)	2,381 (12.2)	528 (2.7)	
**Alcohol consumption before wife pregnancy**
Yes	1,262 (14.8)	5,961 (69.7)	1,096 (12.8)	235 (2.7)	0.717
No	3,658 (14.3)	17,964 (70.3)	3,238 (12.7)	690 (2.7)	

**Table 2:** The distribution of baseline characteristics of fathers enrolled in the cohort by categories of BMI (kg/m^2^).

**Characteristic**	** < 18.5 Underweight (%)**	**18.5 ~23.9** **Normal weight (%)**	**24.0 ~27.9 Overweight (%)**	**≥28.0 Obesity (%)**	***P*-value**
*n*	1,442 (4.2)	18,118 (53.1)	10,592 (31.1)	3,952 (11.6)	
**Age**
< 25 y	206 (8.8)	1,494 (64.1)	420 (18.0)	210 (9.0)	< 0.001
25 ~ 29 y	786 (6.4)	6,990 (56.6)	3,298 (26.7)	1,272 (10.3)	
30 ~ 34 y	372 (3.2)	5,704 (49.7)	3,952 (34.4)	1,448 (12.6)	
≥35 y	78 (1.0)	3,930 (49.4)	2,922 (36.8)	1,022 (12.9)	
**Ethnicity**
Han	1,412 (4.3)	17,480 (53.0)	10,402 (31.5)	3,706 (11.2)	< 0.001
Minority	30 (2.7)	638 (57.8)	190 (17.2)	246 (22.3)	
**Educational level**
Junior high school or Below	120 (2.9)	2,178 (52.6)	1,156 (27.9)	684 (16.5)	< 0.001
High school or Technical school	870 (10.0)	5,424 (62.2)	1,876 (21.5)	544 (6.2)	
Junior collage	388 (2.1)	9,092 (49.5)	6,498 (35.4)	2,384 (13.0)	
Bachelor and above	64 (2.2)	1,424 (49.3)	1,062 (36.8)	340 (11.8)	
**Smoking status before wife pregnancy**
Yes	962 (6.6)	7,260 (49.9)	4,390 (30.2)	1,946 (13.4)	< 0.001
No	480 (2.5)	10,858 (55.6)	6,202 (31.7)	2,006 (10.3)	
**Alcohol consumption before wife pregnancy**
Yes	342 (4.0)	4,110 (48.1)	2,884 (33.7)	1,218 (14.2)	< 0.001
No	1,100 (4.3)	14,008 (54.8)	7,08 (30.2)	2,734 (10.7)	
**Maternal characteristic**
**Age**
< 25 y	57 (3.2)	911 (51.5)	581 (32.8)	220 (12.4)	0.002
25 ~ 29 y	539 (4.5)	6,293 (53.0)	3,716 (31.3)	1,325 (11.2)	
30 ~ 34 y	533 (4.2)	6,933 (54.2)	3,835 (30.0)	1,502 (11.7)	
≥35 y	313 (4.1)	3,981 (52.0)	2,460 (32.1)	905 (11.8)	
**Ethnicity**
Han	1,422 (4.2)	17,885 (53.1)	10,442 (31.0)	3,907 (11.6)	0.581
Minority	20 (4.5)	233 (52.0)	150 (33.5)	45 (10.0)	
**Educational level**
Junior high school or below	93 (3.7)	1,377 (54.1)	779 (30.6)	298 (11.7)	0.354
High school or Technical school	403 (4.2)	5,095 (52.6)	3,094 (31.9)	1,103 (11.4)	
Junior collage	693 (4.4)	8,275 (53.1)	4,794 (30.7)	1,832 (11.7)	
Bachelor and above	253 (4.0)	3,371 (53.8)	1,925 (30.7)	719 (11.5)	
**Parity**
Yes	737 (4.2)	9,411 (53.3)	5,435 (30.8)	2,075 (11.8)	0.530
No	705 (4.3)	8,707 (52.9)	5,157 (31.4)	1,877 (11.4)	
**Family income per month**
≤ 2,500 RMB	816 (13.8)	3,470 (58.9)	1,324 (22.5)	282 (4.8)	< 0.001
2,500–5,000 RMB	420 (2.3)	9,582 (52.6)	5,990 (32.9)	2,214 (12.2)	
>5,000 RMB	206 (2.1)	5,066 (50.6)	3,278 (32.8)	1,456 (14.6)	
**Active smoking before pregnancy**
Yes	0 (0.0)	198 (57.9)	112 (32.7)	32 (9.4)	< 0.001
No	1,442 (4.3)	17,920 (53.1)	10,480 (31.0)	3,920 (11.6)	
**Passive smoking before pregnancy**
Yes	204 (4.1)	2,568 (52.0)	1,596 (32.3)	570 (11.5)	0.215
No	1,238 (4.2)	15,550 (53.3)	8,996 (30.8)	3,382 (11.6)	
**Alcohol consumption before pregnancy**
Yes	8 (1.4)	306 (52.8)	210 (36.2)	56 (9.7)	< 0.001
No	1,434 (4.3)	17,812 (53.1)	10,382 (31.0)	3,896 (11.6)	
**Folic acid consumption before or during pregnancy**
Yes	1,370 (4.2)	17,280 (53.1)	10,136 (31.1)	3,770 (11.6)	0.498
No	72 (4.7)	838 (54.1)	456 (29.5)	182 (11.8)	
**History of adverse pregnancy outcomes**
Yes	179 (3.3)	2,740 (50.9)	1,832 (34.0)	637 (11.8)	< 0.001
No	1,263 (4.4)	15,378 (53.6)	8,760 (30.5)	3,315 (11.5)	
**History of pregnancy complications**
Yes	78 (1.7)	2,218 (48.6)	1,518 (33.2)	754 (16.5)	< 0.001
No	1,364 (4.6)	15,900 (53.8)	9,074 (30.7)	3,198 (10.8)	
**Gestational weight gain range**
Under-GWG group	415 (4.3)	5,254 (54.6)	2,757 (28.6)	1,203 (12.5)	< 0.001
GWG normal group	630 (4.2)	8,105 (53.6)	4,657 (30.8)	1,724 (11.4)	
GWG excessive group	397 (4.2)	4,759 (50.8)	3,178 (34.0)	1,025 (11.0)	
**Pregnancy complications in this pregnancy**
Yes	239 (3.7)	3,392 (52.5)	1,987 (30.7)	847 (13.1)	< 0.001
No	1,203 (4.4)	14,726 (53.3)	8,605 (31.1)	3,105 (11.2)	

### Prevalence of LBW, VLBW, and ELBW

For the entire study population, the prevalence of LBW in offspring was 8.9%, VLBW was 1.9%, and ELBW was 0.4%. For mothers, the prevalence of LBW in offspring of people with normal BMI was lower than that of people with abnormal BMI, in contrast, the prevalence of VLBW and ELBW increased with the increasing weight group, except for ELBW in the obesity group. For fathers, the prevalence of LBW, VLBW, and ELBW in offspring increased with the increasing weight group, except LBW and ELBW in the obesity group (Supplementary Table 1).

### Effect of pre-pregnancy parental BMI on LBW, VLBW, and ELBW

**Figure 1:**
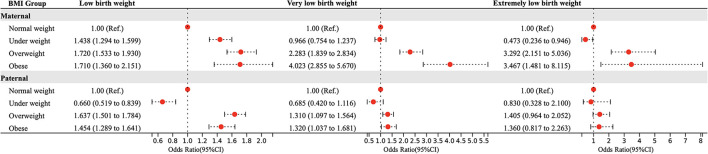
Parental BMI in pre-pregnancy and the risk of LBW, VLBW, and ELBW in offspring. Adjusted for maternal age, ethnicity, educational level, parity, family income per month, active smoking before pregnancy, passive smoking before pregnancy, alcohol consumption before pregnancy, folic acid consumption before or during pregnancy, history of adverse pregnancy outcomes, history of pregnancy complications, gestational weight gain recommendation rage, pregnancy complications in this pregnancy and paternal age, ethnicity, education level, smoking status before wife pregnancy, and alcohol consumption before wife pregnancy.

Figure 1 shows the effects of pre-pregnancy parental BMI on LBW, VLBW, and ELBW using a multivariate logistic regression model after adjusting the confounding factors. For mothers, abnormal weight was a risk factor for LBW (underweight: OR = 1.438, 95% CI = 1.294 ~ 1.599; overweight: OR = 1.720, 95% CI = 1.533 ~ 1.930; obesity: OR = 1.710, 95% CI = 1.360 ~ 2.151), overweight and obesity were risk factors for VLBW as well as ELBW (VLBW: overweight: OR = 2.283, 95% CI = 1.839 ~ 2.834; obesity: OR = 4.023, 95% CI = 2.855 ~ 5.670; ELBW: overweight: OR = 3.292, 95% CI = 2.151 ~ 5.036; obesity: OR = 3.467, 95% CI = 1.481 ~ 8.115), while maternal underweight was a protective factor for ELBW (OR = 0.473, 95% CI = 0.236 ~ 0.946). For fathers, pre-pregnancy overweight and obesity were associated with higher LBW (overweight: OR = 1.637, 95% CI = 1.501 ~ 1.784; obesity: OR = 1.454, 95% CI = 1.289 ~ 1.641) and VLBW (overweight: OR = 1.310, 95% CI = 1.097 ~ 1.564; obesity: OR = 1.320, 95% CI = 1.037 ~ 1.681) risk, and underweight was associated with lower LBW (OR = 0.660, 95% CI = 0.519 ~ 0.839) risk, while no association was found between pre-pregnancy BMI and ELBW.

### The conjunction effect between parental BMI and LBW, VLBW, and ELBW

**Figure 2:**
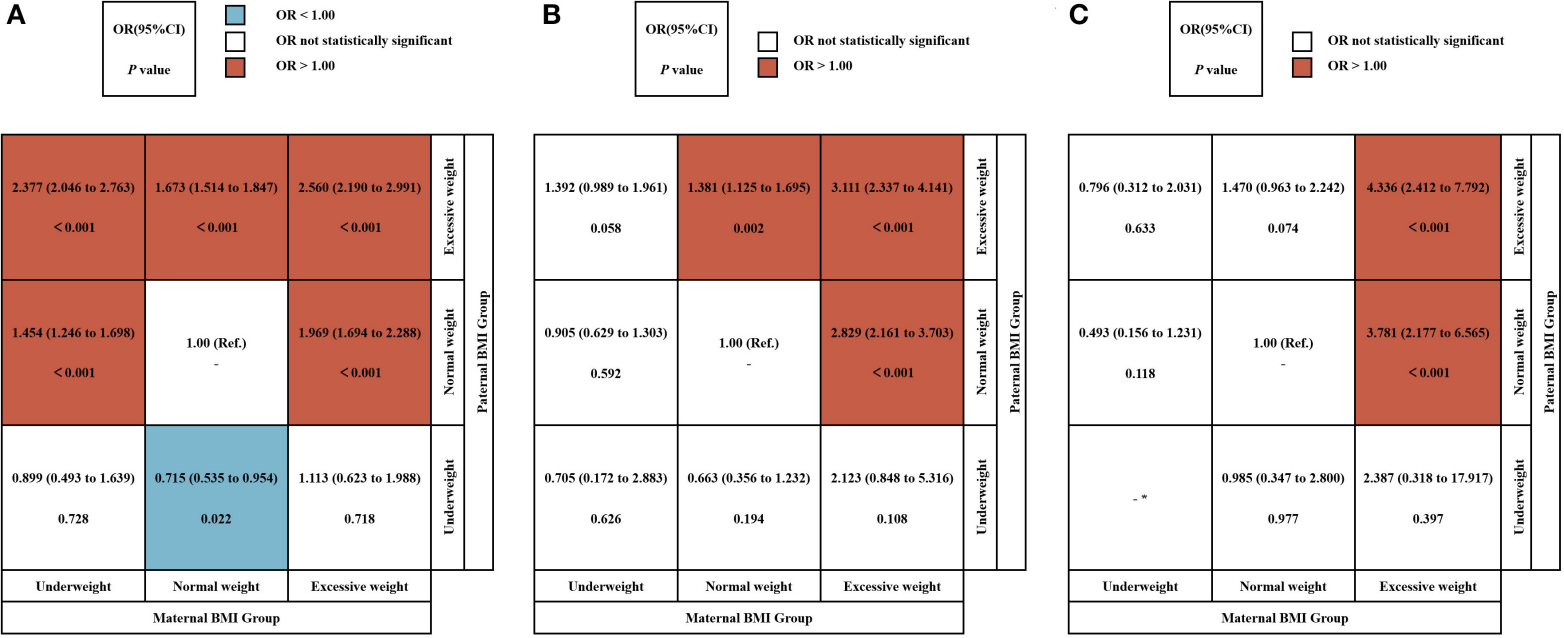
The conjunction effect between parental BMI and LBW, VLBW, and ELBW. The conjunction effect between parental BMI and LBW **(A)**, VLBW **(B)**, and ELBW **(C)**. Adjusted for maternal age, ethnicity, educational level, parity, family income per month, active smoking before pregnancy, passive smoking before pregnancy, alcohol consumption before pregnancy, folic acid consumption before or during pregnancy, history of adverse pregnancy outcomes, history of pregnancy complications, gestational weight gain recommendation rage, pregnancy complications in this pregnancy and paternal age, ethnicity, education level, smoking status before wife pregnancy, and alcohol consumption before wife pregnancy. OR, odds ratio. *Actual frequency is 0.

We used a crossover analysis to explore further the conjunction effect of maternal and paternal pre-pregnancy BMI on offspring LBW, VLBW, and ELBW (Figure 2). Due to the limited number of subjects in the obesity group, the obesity group was combined with the overweight group to create the excessive group, and the paternal and maternal BMI group was combined in a three by three cross-tabulation. After adjusting for confounding factors, the results showed that pre-pregnancy normal-weight mothers and underweight fathers group reduced the risk of LBW in offspring (OR: 0.715, 95% CI = 0.535 ~ 0.954), while pre-pregnancy excessive weight mothers and normal-weight fathers group (LBW: OR: 1.969, 95% CI = 1.694 ~ 2.288; VLBW: OR: 2.829, 95% CI = 2.161 ~ 3.703; ELBW: OR: 3.781, 95% CI = 2.177 ~ 6.565), as well as excessive weight fathers group (LBW: OR: 2.560, 95% CI = 2.190 ~ 2.991; VLBW: OR: 3.111, 95% CI = 2.337 ~ 4.141; ELBW: OR: 4.336, 95% CI = 2.412 ~ 7.792), increased the risk of LBW, VLBW, and ELBW in their offspring. Normal weight mothers and excessive weight fathers group increase the risk of LBW (OR: 1.673, 95% CI = 1.514 ~ 1.847) and VLBW (OR: 1.381, 95% CI = 1.125 ~ 1.695) in their offspring. Underweight mothers and normal weight fathers group, as well as excessive weight fathers group, likewise increase the risk of LBW in their offspring (OR:1.454, 95% CI = 1.246 ~ 1.698; OR: 2.377, 95% CI = 2.046 ~ 2.763).

**Figure 3:**
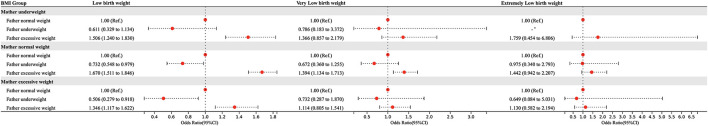
Stratified analysis of paternal BMI in pre-pregnancy and the risk of LBW, VLBW, and ELBW in offspring according to maternal BMI categories. Adjusted for maternal age, ethnicity, educational level, parity, family income per month, active smoking before pregnancy, passive smoking before pregnancy, alcohol consumption before pregnancy, folic acid consumption before or during pregnancy, history of adverse pregnancy outcomes, history of pregnancy complications, gestational weight gain recommendation rage, pregnancy complications in this pregnancy and paternal age, ethnicity, education level, smoking status before wife pregnancy, and alcohol consumption before wife pregnancy.

Based on the crossover analysis, we further performed a stratified analysis by dividing the population into subgroups according to maternal BMI categories, to explore the association between paternal BMI and offspring incidence by controlling for maternal BMI (Figure 3). After adjusting for confounders, the results of multivariate logistic regression showed that in the population with lower maternal BMI, excessive paternal weight was a risk factor for LBW (OR:1.506, 95% CI = 1.240 ~ 1.830); in the population with normal maternal weight, underweight fathers were a protective factor for LBW (OR:0.732, 95% CI = 0.548 ~ 0.979) while overweight was a risk factor for LBW (OR:1.670, 95% CI = 1.511 ~ 1.846) as well as VLBW (OR:1.394, 95% CI = 1.134 ~ 1.713); for the population with overweight mothers, underweight fathers were also a protective factor (OR:0.506, 95% CI = 0.279 ~ 0.918) and overweight was a risk factor for LBW (OR:1.346, 95% CI = 1.117 ~ 1.622).

### Dose-response relationship between pre-pregnancy BMI and the risk of LBW, VLBW, and ELBW

**Figure 4:**
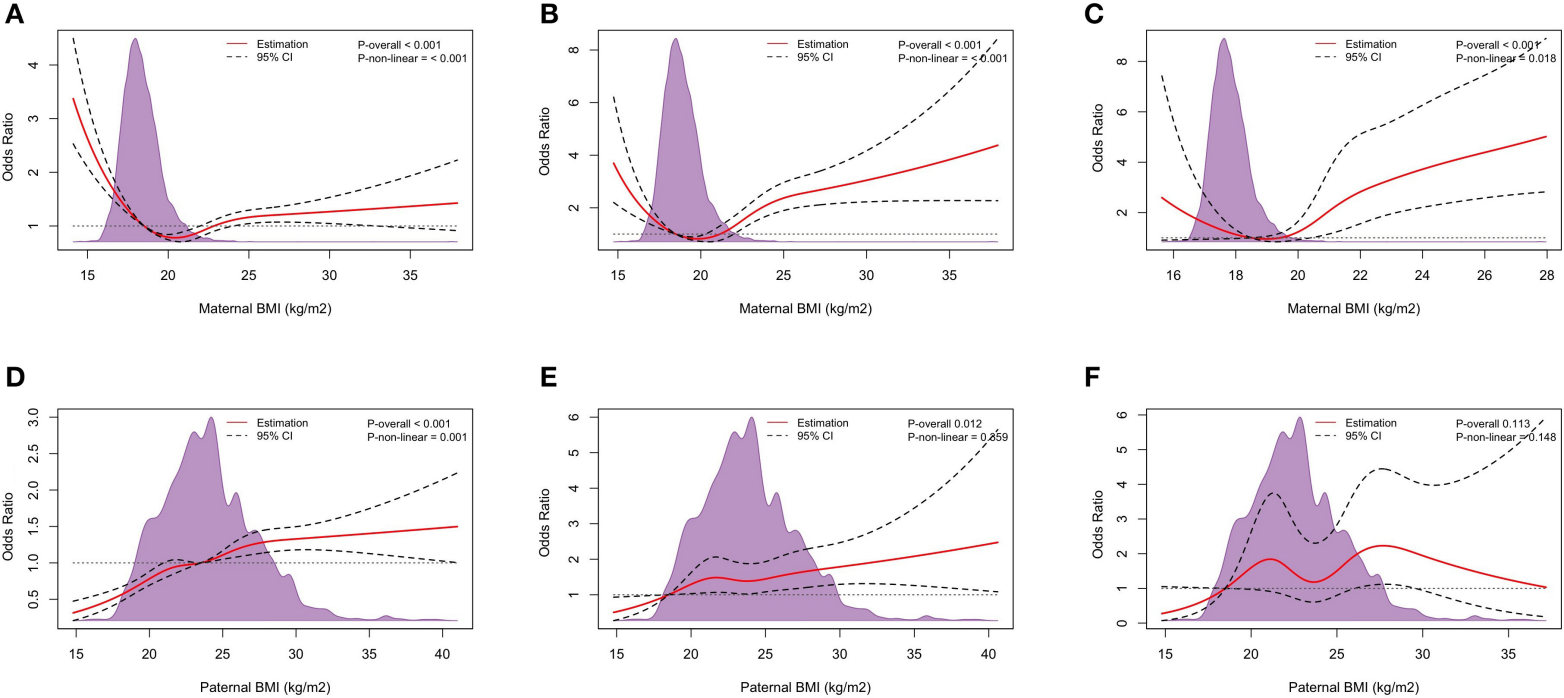
Dose-response relationship. The Dose-response relationship between maternal pre-pregnancy BMI and LBW **(A)**, VLBW **(B)**, and ELBW **(C)**, and paternal pre-pregnancy BMI and LBW **(D)**, VLBW **(E)**, and ELBW **(F)**. Adjusted for maternal age, ethnicity, educational level, parity, family income per month, active smoking before pregnancy, passive smoking before pregnancy, alcohol consumption before pregnancy, folic acid consumption before or during pregnancy, history of adverse pregnancy outcomes, history of pregnancy complications, gestational weight gain recommendation rage, pregnancy complications in this pregnancy and paternal age, ethnicity, education level, smoking status before wife pregnancy, and alcohol consumption before wife pregnancy.

Figure 4 shows the dose-response relationship between parental pre-pregnancy BMI and the risk of LBW, VLBW, and ELBW. The RCS logistic regression showed that except for the association between paternal BMI and the risk of VLBW (*P*_non − linear_ > 0.05), the other associations were non-linear dose-response relationships (*P*_non − linear_ < 0.05). There was no dose-response relationship between paternal and the risk of ELBW (*P*_overall_ > 0.05).

## Discussion

In this study, we found that parental pre-pregnancy body mass index was associated with the risk of low birth weight in the offspring. Maternal pre-pregnancy overweight and obesity were associated with higher LBW, VLBW, and ELBW risk, in comparison underweight was associated with higher LBW and lower ELBW risk. Paternal pre-pregnancy overweight and obesity were associated with higher LBW and VLBW risk, and underweight was associated with lower LBW risk, while no association was found between pre-pregnancy BMI and ELBW. The conjunction effect analysis showed that parents who were both excessive-weights in pre-pregnancy BMI, and overweight mothers with normal-weight fathers before pre-pregnancy, were more likely to have offspring with LBW, VLBW, and ELBW. In contrast, their offspring were at less risk of LBW for normal-weight mothers and underweight fathers. Stratified analyses supported the above findings, after controlling for maternal BMI in the study population, excessive-weight fathers increased the risk of LBW; in those with normal-weight mothers, the offspring of underweight fathers had a lower risk of LBW. Dose-response relationship existed between parental pre-pregnancy and LBW, VLBW, and ELBW, except for paternal BMI and ELBW.

Our results showed that maternal pre-pregnancy abnormal BMI increases the risk of LBW, VLBW, and ELBW in the offspring, which confirms the results of previous similar studies (26–29). Low maternal BMI may result from chronic inadequate energy intake and malnutrition, which reduces fat stores and impairs visceral and somatic cell protein status (30). In addition, a prolonged pre-pregnancy low BMI state will increase the energy required during pregnancy and the mother will make the energy deficit in early pregnancy insufficient to meet the substrates needed to support fetal tissue growth (30). The results of the meta-analysis suggested that underweight women had an increased risk of LBW (31, 32), as well as an increased risk of infants with VLBW and a tendency to increase ELBW (32). Interestingly, in our study, it was observed that low maternal BMI was a protective factor for ELBW. However, the study by Nakanishi et al. (26) showed no association between low BMI and ELBW due to the small number of outcomes of ELBW in this study. Therefore, the association between maternal wasting and ELBW needs to be further investigated. Obesity is a common problem in women of reproductive age, it is well-known for its negative impact on reproductive physiology. Obesity and overweight involve abnormal and excessive fat accumulation that negatively affects health status, as well as the presence of menstrual irregularities, ovulation disorders, and endometrial lesions (33). The effects of obesity on female reproductive function are mainly attributed to endocrine mechanisms (34): interference with neuroendocrine and ovarian functions causing reduced ovulation and severe neurological disorders of the hypothalamic-pituitary-ovarian axis (35), among others, as well as an increased risk of adverse pregnancy outcomes and poor neonatal due to the body's obesity status (36). Gestational weight gain (GWG) is also one of the factors influencing LBW. Some studies have suggested that the discriminative performance of GWG with adverse pregnancy outcomes was lower, and pre-pregnancy BMI was stronger associated with adverse pregnancy outcomes than the amount of GWG (37), so in this study, we adjusted for it as a confounding factor.

In previous studies, most researchers have focused on the influence of maternal factors and have not considered paternal factors and the possible effects of couples acting together. Our study reported that paternal pre-pregnancy overweight and obesity were a risk factor for LBW and VLBW, and underweight was a protective factor for LBW, there was no relationship between paternal BMI and ELBW. After stratification of maternal BMI, excessive paternal BMI was a risk factor for LBW in all maternal BMI categories, and excessive paternal BMI also increased the incidence of VLBW in those with normal maternal BMI, whereas paternal underweight was a protective factor for LBW in those with non-underweight maternal categories. A study by He et al. (38) reported that vs. fathers with normal BMI, high BMI is a risk factor for LBW, which was consistent with our results. However, the current effects on paternal pre-pregnancy BMI appear to be uncertain and contradictory, the results of the meta-analysis reported a non-significant relationship between paternal weight or BMI and LBW (39, 40), and a cohort study reported paternal higher BMI could decrease the rate of LBW, their association needs to be verified in the future in a larger multi-ethnic population. Men with low body weight have reduced sperm concentration, total sperm number and total motile sperm count compared to men with normal BMI (20). Mechanisms by which obesity may affect spermatogenesis include thermal effects, hyperestrogenism, hypogonadotropic hypogonadism, diabetes, sexual dysfunction, and sperm epigenetic perturbations (41), in addition to the indirect effects of obesity on the father that may be transmitted to the offspring through genetic and epigenetic alterations in germ cell DNA, with detrimental effects on the offspring (42, 43).

Theoretically, an infant's birth weight is influenced by a combination of genetic factors of the mother and father as well as environmental factors. Mutsaerts et al. (18) suggested that paternal lifestyle factors do not exert an independent effect on the investigated outcomes, while maternal BMI influences the risk of pregnancy complications and perinatal outcomes. Retnakaran et al. (17) concluded that an increase in both paternal pre-pregnancy BMI increased infant birth weight, with maternal pre-pregnancy BMI explaining 6.2% of the difference in birth weight while the father explained only 0.7%. Our study preliminarily uncovered the effect of the conjunction of parental BMI on LBW, excessive weight mothers and normal weight or excessive weight fathers are more likely to result in LBW offspring, and such effects are also observed in the offspring of VLBW and ELBW. In addition, we observed that the normal weight mother-underweight father group was protective for LBW. In a multivariate logistic analysis, we found that underweight fathers were protective for LBW (OR = 0.660, 95% CI: 0.519 ~ 0.839). In a further stratified analysis based on the conjunction effect, the protective effect of underweight fathers on LBW remained in the normal weight mother categories. This is an intriguing finding for the prevention of LBW, but given that the existing studies on the effect of paternal BMI on LBW have failed to clearly elucidate, this finding and how to quantify the magnitude of the effect of parental BMI on offspring morbidity need further investigation in the future.

We also looked at the dose-response relationship between parental pre-pregnancy BMI and the risk of LBW, VLBW, and ELBW. Our results showed a dose-response relationship between the associations except for the absence of a dose-response relationship between paternal BMI and ELBW. Nakanishi et al. (26) found that the dose-response relationship between the severity of low pre-pregnancy BMI and LBW was found only in the low BMI range, and the association between maternal low BMI and VLBW and ELBW was not statistically significant, but the U-shaped relationship was observed in the RCS. There are no studies on the association of paternal BMI with LBW, VLBW, and ELBW, so more research on the dose-response relationship about this topic is needed.

The novelty of this study is significant. First, this study comprehensively explored the association of LBW, VLBW, and ELBW with paternal and maternal BMI, as most previous studies on low birth weight have focused only on infants with birth weight < 2,500 g and have not comprehensively explored VLBW (birth weight < 1,500 g) as well as ELBW (birth weight < 1,000 g) infants. Second, previous studies on pregnancy outcomes have focused on maternal factors. This study further discussed the role of maternal factors and combined parental effects in the occurrence of adverse pregnancy outcomes. Third, we explored the dose-response relationship between maternal and paternal pre-pregnancy BMI and the risk of LBW, VLBW, and ELBW, which has received little attention in previous studies.

This study has some strengths and limitations. First, a significant strength is the large sample size and prospective data collection, and the prospective cohort design minimizes recall bias and ensures the reliability of the study. Second, the use of pre-pregnancy and early pregnancy as the focus periods of this study also reinforced the reliability of the results. Furthermore, convenient and effective communication methods were established between researchers and participants, including telephone and WeChat, which helped reduce the rate of loss to follow-up (1.7% in this study). For limitations, participants were asked to recall their pre-pregnancy body weight and height, this produces a degree of bias. Second, our study was a single-center cohort study in which we recruited participants from one hospital and no other regional hospital. This limitation may lead to subsequent problems, including the representativeness of the sample and the generalization of the study results. The results of this study may not reflect those of other institutions across the country, and similar studies need to be conducted on other maternal populations in China in future studies. In addition, because of the limited sample size of ELBW, which reduces the precision of the odds ratio, studies of ELBW still need to be conducted in larger populations.

## Conclusion

In this study, we found that parental pre-pregnancy BMI was associated with the risk of low birth weight in offspring. Maternal pre-pregnancy overweight and obesity were associated with a higher risk of LBW, VLBW, and ELBW, while underweight was associated with a higher risk of LBW and a lower risk of ELBW. Paternal pre-pregnancy overweight and obesity were associated with a higher risk of LBW and VLBW, while underweight was associated with a lower risk of LBW. Parents who were both excessive-weights in pre-pregnancy BMI, as well as overweight mothers and normal-weight fathers before pre-pregnancy, were more likely to have offspring with LBW, VLBW, and ELBW. Dose-response relationship existed between parental pre-pregnancy and LBW, VLBW, and ELBW, except for paternal BMI and ELBW. Our study highlights the role of the father and the importance of the conjunction role of the couple in the incidence of LBW. However, given some limitations of this study, more detailed and large-scale studies are needed.

## Data availability statement

The data performed and/or analyzed in this study are available on reasonable request from the corresponding author.

## Ethics statement

The study received approval from the Ethics Committee of Xiangya School of Public Health Central South University (No: XYGW-2018-36) and was conducted based on the Helsinki declaration. The study protocol has been registered with the Chinese Clinical Trial Registry (No: ChiCTR1800016635). We obtained written informed consent from all participants.

## Author contributions

DX and JQ: conceptualization, funding acquisition, and supervision. JW: data curation and writing—original draft. TW, YL, XS, and MS: formal analysis. JW, JS, TZ, QC, and ML: investigation. JW and TW: methodology. SZ, PH, and PZ: resources. JW, TW, and JS: software. JW, DX, and JQ: visualization and writing—review & editing. All authors have read and agreed to the published version of the manuscript.

## Funding

The study was supported by the National Natural Science Foundation Program of China (82073653 and 81803313), Hunan Provincial Key Research and Development Program (2018SK2063 and 2018SK2062), Hunan Provincial Distinguished Young Scholars Foundation (2022JJ10087), Hunan Provincial Science and Technology Talent Support Project (2020TJ-N07), Open Project from NHC Key Laboratory of Birth Defect for Research and Prevention (KF2020006), China Postdoctoral Science Foundation (2020M682644), the Foundation of the Ministry of Health of Hunan Province, China (202212034013), Natural Science Foundation of Hunan Province (2022JJ40208), the Key Program of Maternal and Child Health Hospital of Hunan Province (2021RX05), the Fundamental Research Funds for the Central Universities of Central South University (2022ZZTS0289), and the Postgraduate Scientific Research Innovation Project of Hunan Province (CX20220371).

## Conflict of interest

The authors declare that the research was conducted in the absence of any commercial or financial relationships that could be construed as a potential conflict of interest.

## Publisher's note

All claims expressed in this article are solely those of the authors and do not necessarily represent those of their affiliated organizations, or those of the publisher, the editors and the reviewers. Any product that may be evaluated in this article, or claim that may be made by its manufacturer, is not guaranteed or endorsed by the publisher.

## Supplementary material

Supplementary Figure 1 Flow chart showing the process of participant recruitment.

Supplementary Table 1 Prevalence (% and 95% CI) of LBW, VLBW, and ELBW in offspring by parental pre-pregnancy BMI.
