# Supplementary figures and images for: Parental pre-pregnancy body mass index and risk of low birth weight in offspring: A prospective cohort study in central China
Source: Front Public Health. 2022 Nov 30;10:1036689. doi: 10.3389/fpubh.2022.1036689 (PMC9748483; doi:10.3389/fpubh.2022.1036689)

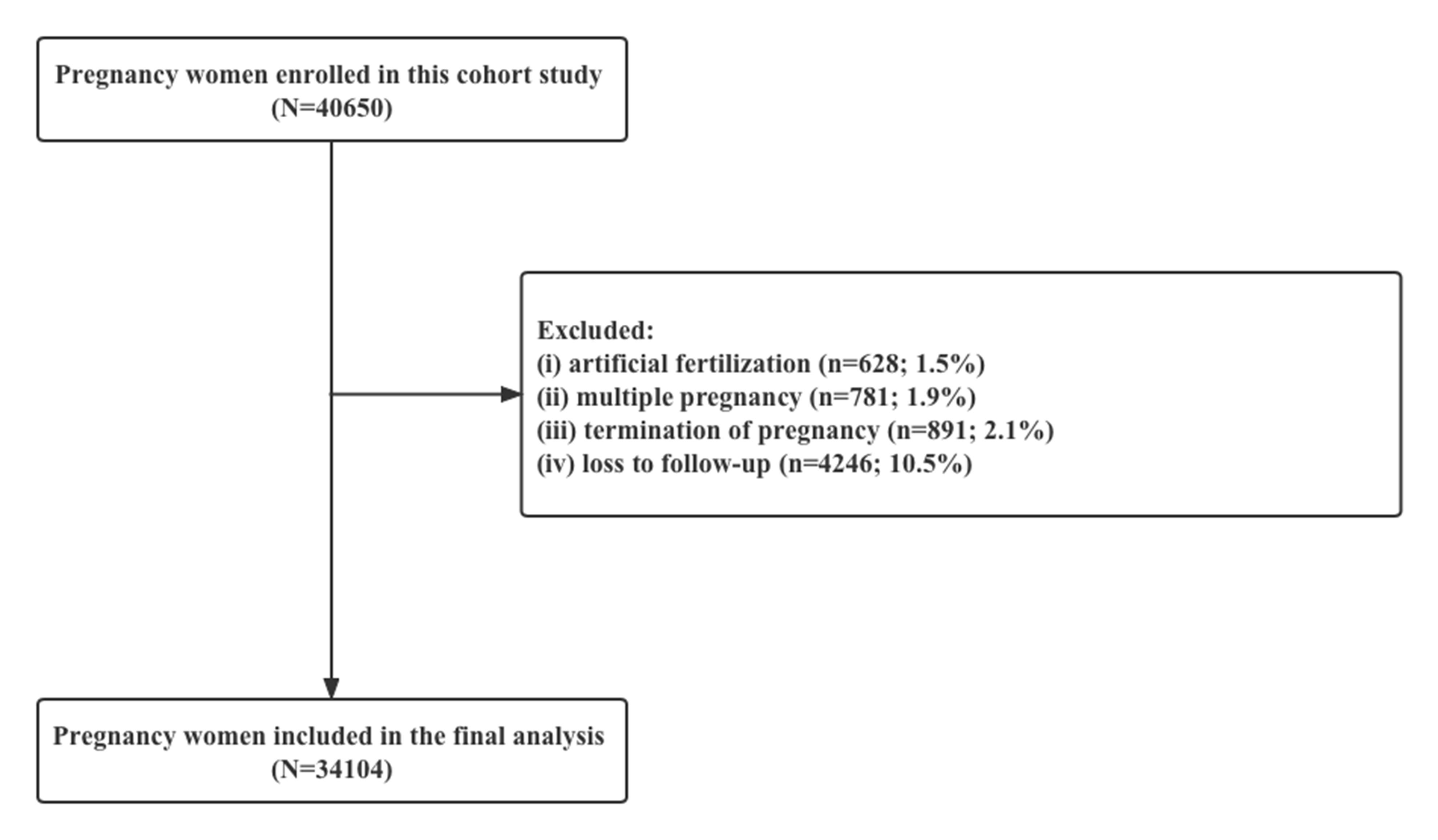

Supplement: Supplementary Figure 1 — Flow chart showing the process of participant recruitment. [file Image_1.PNG]
