# Supplementary material for: Parental pre-pregnancy body mass index and risk of low birth weight in offspring: A prospective cohort study in central China
Source: Front Public Health. 2022 Nov 30;10:1036689. doi: 10.3389/fpubh.2022.1036689 (PMC9748483; doi:10.3389/fpubh.2022.1036689)
Supplement: Supplementary Table 1 — Prevalence (% and 95% CI) of LBW, VLBW, and ELBW in offspring by parental pre-pregnancy BMI. [file Table_1.DOCX]

Table S1 Prevalence (% and 95%CI) of LBW, VLBW, and ELBW in offspring by parental pre-pregnancy BMI

| Pre-Pregnancy BMI (kg/m^2^) | LBW % (95%CI) | VLBW % (95%CI) | ELBW % (95%CI) |
| --- | --- | --- | --- |
| Total | 8.9 (8.6 to 9.2) | 1.9 (1.7 to 2.0) | 0.4 (0.3 to 0.5) |
| Maternal |  |  |  |
| Under weight (<18.5) | 11.2 (10.3 to 12.1) | 1.6 (1.3 to 2.0) | 0.2 (0.1 to 0.3) |
| Normal weight (18.5~23.9) | 8.0 (7.6 to 8.3) | 1.7 (1.5 to 1.8) | 0.4 (0.3 to 0.5) |
| Overweight (24.0~27.9) | 10.9 (9.9 to 11.8) | 2.9 (2.4 to 3.4) | 0.7 (0.5 to 1.0) |
| Obesity (≥28.0) | 10.3 (8.3 to 12.2) | 4.5 (3.2 to 5.9) | 0.6 (0.1 to 1.2) |
| Paternal |  |  |  |
| Under weight (<18.5) | 5.5 (4.4 to 6.7) | 1.2 (0.7 to 1.8) | 0.3 (0.1 to 0.7) |
| Normal weight (18.5~23.9) | 7.6 (7.2 to 8.0) | 1.7 (1.5 to 1.9) | 0.4 (0.3 to 0.5) |
| Overweight (24.0~27.9) | 10.9 (10.3 to 11.4) | 2.1 (1.8 to 2.4) | 0.5 (0.3 to 0.6) |
| Obesity (≥28.0) | 10.6 (9.6 to 11.5) | 2.3 (1.8 to 2.8) | 0.5 (0.3 to 0.7) |

BMI= body mass index; LBW= low birth weight; VLBW= very low birth weight; ELBW= extremely low birth weight; 95%CI= 95% confidence interval.
